# A Diagnostic Dilemma: Is It Factitious Disorder With Nonepileptic Seizure or Malingering With Nonepileptic Seizure?

**DOI:** 10.7759/cureus.39197

**Published:** 2023-05-18

**Authors:** Badar Sabeen, Temilola Majekodunmi, Abdulhusein Kapasi, Sherrie Bieniek, David Leszkowitz

**Affiliations:** 1 Department of Addiction Medicine (Palm Springs Campus), Larkin Community Hospital, Hialeah, USA; 2 Department of Research and Academic Affairs, Larkin Community Hospital, South Miami, USA; 3 Department of Psychiatry, Larkin Community Hospital, South Miami, USA; 4 Department of Addiction Medicine, Larkin Community Hospital, South Miami, USA

**Keywords:** disorder, seizure, nonepileptic, malingering, factitious, functional

## Abstract

In reality, the lines between factitious disorder, functional disorder, and malingering are quite blurred. In factitious disorder and malingering, patients consciously and deliberately create false medical and/or psychiatric symptoms for self-gain, often approaching multiple healthcare facilities to evade detection. Although the factitious disorder is pervasive, and the literature lacks accurate and consistent information, comorbidity with nonepileptic seizure (NES, a component of functional disorder) is quite commonly documented. In our case, the patient feigned multiple symptoms including two seizures and a shoulder dislocation to gain access to opioids. The clinical picture was only significant for alcohol withdrawal, aspiration pneumonia (possibly intubation vs. NES-related), and self-induced shoulder dislocation. Generally, management of these disorders should involve multiple specialties, multiple approaches, and identifying the triggering and comorbid psychological disorders, such as abandonment issues, personality disorders, physical or emotional abuse, anxiety, depression, stress, and substance use. Blindly approaching patients with a factitious disorder or malingering will not lead to any productive outcomes. Perhaps, creating a patient database could help reduce futile efforts while providing patients with the required help. This case report describes the presentation, diagnosis, management, and outcomes related to a patient with NES, engaging the reader to decipher the most appropriate diagnosis.

## Introduction

Knowledge of the etiology of factitious disorder and its prevalence is limited due to the scarcity of large-scale studies on the subject. The condition accounts for ~0.8-1% of psychiatric consults and, according to the literature, it is more prevalent among females, adolescents/middle-aged individuals, single/unmarried people, and healthcare workers [1-3]. Triggers such as childhood trauma, neglect, abuse, abandonment, truncated social attachments, and the death of a loved one may contribute to its etiology [2,4]. Treatment relies heavily on psychotherapy. We discuss a case involving the overlap and variation in the presentation of functional disorder vs. factitious disorder, and factitious disorder vs malingering. We delineate the presentation, diagnosis, and management of a patient with recurrent nonepileptic seizures (NES). We also discuss possible collaborative measures for the early diagnosis of these cases.

## Case presentation

A 30-year-old Caucasian woman voluntarily presented to the Emergency Department complaining of six to eight hours of tremors, nausea, and severe anxiety, as well as insomnia for three consecutive nights after opioid and alcohol use. Seemingly unperturbed by her symptoms, she reported a nine-year history of daily drug use (fentanyl, benzodiazepines, and crack cocaine) together with occasional beer intake. She had received several opioid detoxification therapies in the past, and undergone multiple ICU admissions for withdrawal seizures, but she denied any history of psychiatric illnesses.

Following an unremarkable physical examination, a Clinical Institute Withdrawal Assessment-Alcohol (CIWA) score was obtained, together with a blood and urine drug screen (UDS). Her CIWA score was 20, signifying severe alcohol withdrawal, and UDS was positive for cocaine (Table 1). A diagnosis of severe alcohol withdrawal symptoms was made, and the patient was admitted for medical management.

**Table 1:** Laboratory values on admission and the mean and range for the entire hospital stay of nine days The panel includes a complete blood count (CBC), complete metabolic profile (CMP), and urine drug screen (UDS) WBC: white blood cell count; RBC: red blood cell count; Hb: hemoglobin; HCT: hematocrit; MCV: mean corpuscular volume; RDW-SD: red cell distribution width-standard deviation; PLT: platelets; MPV: mean plasma volume; BUN: blood urea nitrogen; ALP: alkaline phosphatase; ALT: alanine aminotransferase; AST: aspartate aminotransferase; CK: creatinine kinase; GFR: glomerular filtration rate; MTD: methadone; PCP: phencyclidine; THC: tetrahydrocannabinol

Labs	On admission	Mean	Range
WBC (cells/L)	8.9	9.3	5.7–14.4
RBC (million cells/mcL)	4.3	3.9	3.53–4.28
Hb (g/dL)	12.3	11.4	10.1–12.4
HCT (%)	35.1	32.9	29.7–36.9
MCV (fL)	82.0	84.3	82–88
RDW-SD (fL)	39.9	41.7	39.4–44.4
PLT (x 10^9^/L)	341.0	330.4	263–480
MPV (fL)	9.4	9.6	9.3–10.1
Neutrophils (%)	60.1	72.4	55.9–90.4
Lymphocytes (%)	28.8	18.1	4.8–31.3
Monocytes (%)	5.7	5.0	3.9–6
Eosinophils (%)	4.3	4.1	0–11.3
Sodium (mmol/L)	137	137.7	135–145
Potassium (mmol/L)	3.5	3.6	3.3–4.2
Chloride (mmol/L)	104.0	107.0	104–109
Carbon dioxide (mmol/L)	27.0	21.0	16–27
Anion gap (mEq/L)	9.5	12.2	9.3–16.2
Glucose (mg/dL)	96.0	94.1	72–100
BUN (mg/dL)	9.0	6.3	3–9
Creatinine (mg/dL)	0.6	0.5	0.45–0.63
BUN-creatinine ratio (mg/dL)	14.0	12.4	6–18
Calcium (mEq/L)	8.2	7.8	7.3–8.2
Albumin (g/dL)	3.9	3.3	3–3.9
Total protein (g/dl)	6.2	6.0	5.4–6.7
ALP (IU/L)	75.0	71.7	65–76
ALT (IU/L)	21.0	14.7	11–21
AST (IU/L)	24.0	23.8	16–32
Total bilirubin (mg/dL)	0.5	0.5	0.3–0.8
Osmolarity (osmols/L)	263.0	261.7	259–265
CK (U/L)	-	42.0	-
GFR (mL/min)	111.0	144.3	111–187
Magnesium (mEq/L)	-	1.8	1.7–1.8
Phosphorus (mg/dL)	-	2.8	<2.2–2.8
Alcohol (mmol/L)	10.0	10.0	-
Amphetamine	Negative	-	-
Barbiturates	Negative	-	-
Benzodiazepines	Negative	-	-
Cocaine	Positive	-	-
MTD	Negative	-	-
Opiates	Negative	-	-
PCP	Negative	-	-
THC	Negative	-	-

Admission and hospital care

**Figure 1:**
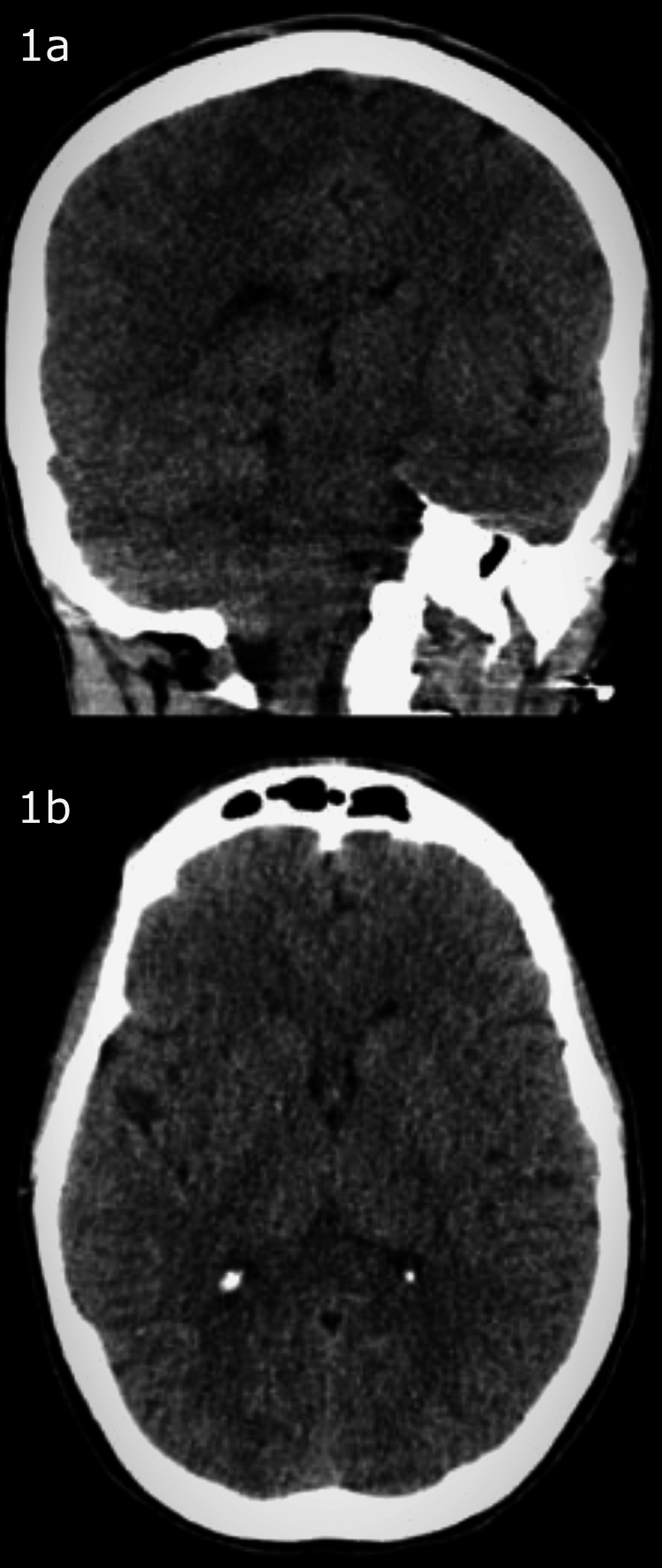
CT brain without contrast 1a: coronal view without contrast showing normal integrity of bony, soft tissue, and vascular structures. 1b: horizontal view without contrast showing normal integrity of bony, soft tissue, and vascular structures CT: computed tomography

A few hours into admission, a rapid response was called, with the primary team and ICU team in attendance; the patient was awake but actively seizing. Three doses of lorazepam 2 mg IV were pushed q10 min, and the patient was transferred to the ICU where she received Divalproex sodium 20 mg/kg IV drip (in two divided doses) over the next 24 hours for substance-induced withdrawal seizure. Sedation and intubation were performed to protect the airway. Following evaluation by the consulted neurologist, a CT brain (Figures 1a, 1b) and electroencephalogram (EEG) were performed, which were both unremarkable. On day two, the patient’s mother called to inform the care team that her daughter had been intubated multiple times in the past after fabricating seizures. With this piece of information, the providers decided to observe the patient; extubation was planned for the following day.

After extubation on day three, tachypnea, tachycardia, and fever ensued and the patient was placed on bilevel positive airway pressure (BiPAP) support. A pneumonia workup (CXR, chest CT, blood cultures) and echocardiogram were all normal except for the chest CT which showed possible aspiration. Aspiration management was started, and the patient was transferred to the medical floor for medical management. On day six, the CIWA score was 5; the patient admitted to feigning her symptoms and requested help in quitting substance use. By day eight, self-induced tremors and slurred speech in addition to her ongoing anxiety and agitation were observed, thus warranting an involuntary hold for the patient’s safety. At this point, a diagnosis of factitious disorder was made. On day nine, while preparing for transfer to a more specialized psychiatric facility, the patient complained of left shoulder pain. Following orthopedic clearance with a negative X-ray, the patient was transferred to an inpatient psychiatric facility.

On arrival at the inpatient psychiatric hospital, the patient exaggerated her left shoulder pain, resulting in a transfer to a different mental health center with emergency room (ER) service; following another X-ray on arrival, a left shoulder dislocation was identified, and the patient proceeded to display NES. As a result, the ER doctor sedated and intubated her once again; however, a repeat EEG showed no seizure activity. The patient was diagnosed with a dislocated left shoulder and NES, admitted for four days, and was stabilized. Once stable, she was transferred back to our hospital where she received a dose of naltrexone to prevent relapse and psycho-social management was started.

The Marchman Act (a Florida law that enables relatives of patients with substance use disorder to obtain involuntary and voluntary treatment) was filed by the patient’s mother, enabling the patient to receive needed care, and she was successfully transferred to an inpatient rehabilitation facility. One month later, the patient received a follow-up call in rehabilitation and reported receiving a second dose of naltrexone with marked improvement in cravings and abstinence. Unfortunately, by the time of her second follow-up, the patient had relocated, and her phone and email were inaccessible.

## Discussion

This case highlights the challenges physicians face in diagnosing NES. Our patient presented with complaints of alcohol and opioid withdrawal symptoms to the ER, which progressed to seizure within 24 hours. This peculiar presentation prompted treatments for opioid withdrawal-induced seizures, and then for pneumonia, shoulder dislocation, and intubation. Although the providers in this case suspected factitious disorder, it was difficult to ignore the initial CIWA score of 20 (even though it is a highly subjective test typically obtained via mobile apps in our hospital setting), alongside other clinical symptoms, such as fever and lung consolidations as seen on CT chest, and even the self-induced left shoulder dislocation.

In malingering, patients intentionally feign or exaggerate presenting symptoms for some tangible or observed gain. However, these patients retain enough insight not to self-inflict harm/injury. If at all self-harm is inflicted, it tends to be minimal at best. Additionally, clinicians have found that these patients usually have a low level of comorbidities compared to those with factitious disorder [5]. Interestingly, our patient admitted to drug seeking; however, the extent of her self-inflicted harm did not seem to fit this diagnosis quite well. The patient went to great lengths to fabricate her symptoms unperturbed by the dangers she placed herself in, all for the "pursuit of opioids".

Conversely, patients with factitious disorder present with repeated and intentionally fabricated symptoms for no obvious gain (e.g., for self-gratification from receiving care, or deceiving health professionals). Factitious disorder has been found prevalent in young females under the age of 40 years, healthcare workers, and substance abusers who self-inflict harm on themselves [5]. The literature shows that these patients usually have psychological comorbidities such as anxiety, depression, and personality disorder [6]. There is no saying to what length a patient with factitious disorder would go to obtain self-gratification. They tend to relocate frequently to evade detection but sometimes open up when help is desired. Although our patient appeared to seek help, a change of address and phone on the follow-up visit suggested otherwise.

In this case, it was fascinating to see how a sedated patient could intentionally fabricate clinical symptoms. Perhaps her substance abuse involved sedatives, giving her a higher tolerance threshold than most, or her pre-programmed designs pervaded the central nervous system (CNS) to produce physical symptoms. One could infer that the patient had an element of functional disorder (aka conversion disorder), whereby motor and sensory functions are disordered based on non-volition and are disabling to the patients [7]. There seems to be some discrepancy in the literature on functional disorders. Per the Diagnostic and Statistical Manual of Mental Disorders, Fifth Edition (DSM-5)/the International Classification of Diseases, Tenth Revision (ICD-10), NES is classified under functional disorders; however, it is not a diagnosis of exclusion [8]. The literature describes NES as a rare presenting symptom of both functional and factitious disorders [8,9]; patients with factitious disorder usually have comorbidities (such as chronic anxiety, prior sexual abuse, and a generally lower QOL), and may experience stress/conflicts but have the insight to show the willingness to change [8,10]. However, in functional disorder, patients are unable to translate reason into action, and are therefore unable to overcome their symptoms: inferring that although they are willing to change, they cannot in fact enact the change [7]. Based on our patient’s self-admission to seeking opioids, the willingness to change, and the sheer difficulty in proving unintentionality, the likelihood that our patient suffered from an underlying functional disorder seems quite dismal.

A proper diagnosis of any one of these disorders - functional disorder, factitious disorder, or malingering - involves a multidisciplinary, collaborative approach (including proper physician training in diagnosis and communication) [11,12]. There are currently no known biomarkers to help in the diagnostic process. One study conducted to identify biomarkers in NES was not successful [13]. The management of psychogenic seizures is multidisciplinary and involves psychotherapy, cognitive behavioral therapy (CBT), mindfulness-based intention treatment, psychoeducation, medical intervention, and shock therapy among others. Studies have shown a reduction in NES in about 14-23% of untreated patients, compared to a 47% reduction in patients who received treatment. Psychotherapy alone reduced the incidence of NES by half in about 80% of patients within 3-52 weeks of starting the treatment [14,15].

From a practical and financial standpoint, factitious disorder and malingering can prove to be costly to both the patient and the healthcare system as a whole. Hospitalization cost alone for one reported case was about $250,000 [16], while another case recorded a total cost of care in excess of $1.1 million [16,17]. Currently, there is no estimate on the prevalence of factitious disorder or malingering. Perhaps the creation of a database could help solve this mystery while empowering physicians to make early diagnoses to provide adequate treatment and reduce the reliance on unnecessary methods and procedures. In this case, it took the primary care team eight days to make a diagnosis of factitious disorder and took professionals from four different specialties (Primary care, Addictive Addiction Medicine, Psychiatry, and Neurology) to diagnose NES in this patient; and three additional specialties (Anesthesiology, Orthopaedics, and Emergency Medicine) were involved in properly managing this patient’s symptoms. A tangible key to ensuring continued care without engaging in fruitless endeavors is efficient documentation and communication. As in this case, where the patient was transferred to different facilities, it would only be expedient to properly document and verbally communicate the patient’s history to the receiving party. It is unsure whether proper documentation and communication occurred between the large inpatient psychiatric center and the ER. However, one could deduce that the ER doctor was not properly informed about the patient’s history, and hence a second re-intubation was performed at the presentation of NES.

## Conclusions

This case report highlights the difficulties in diagnosing NES while managing other vague symptoms in a patient with substance abuse disorder. It illustrates how early diagnosis, proper documentation, effective transference of information, identification of triggering/underlying causes, and interdisciplinary medical management need to work synchronously to avoid fruitless endeavors while improving patient safety within clinical settings. Unfortunately, the patient was eventually lost to further follow-up. When faced with discrepancies in clinical findings and patient presentation (especially in the context of the ongoing opioid crises), physicians should always maintain a high index of suspicion for one of these three differentials: functional disorder vs. factitious disorder vs. malingering, being mindful that overlap among these diagnoses might occur.
